# From VALID principles to community standards in computational biology

**DOI:** 10.1002/qub2.70060

**Published:** 2026-11-04

**Authors:** Yue Cao, Jean Y. H. Yang

**Affiliations:** ^1^ Sydney Precision Data Science Centre The University of Sydney Sydney New South Wales Australia; ^2^ Charles Perkins Centre The University of Sydney Sydney New South Wales Australia; ^3^ School of Medical Sciences The University of Sydney Sydney New South Wales Australia; ^4^ School of Mathematics and Statistics The University of Sydney Sydney New South Wales Australia

**Keywords:** algorithm evaluation, artificial intelligence, community standards

Artificial intelligence (AI) is rapidly reshaping biological research, offering new capabilities in protein modelling, medical imaging, and single‐cell genomics. The emerging challenge is not simply to improve model performance, but to determine whether these advances produce reliable biological knowledge. High predictive performance does not necessarily imply biological insight, nor does it guarantee that a method will generalise across datasets, platforms or populations. Rigorous evaluation is therefore essential for translating AI methods into a real‐world biological utility.

The perspective by Khang et al. directly addresses this challenge with the VALID framework [1]. They argue that evaluation should consider more than model accuracy, including whether an analysis is biologically meaningful, methodologically sound, able to generalise, transparent in its preprocessing, and suitable for its intended use. The VALID framework also highlights common problems such as data leakage, shortcut learning and inappropriate metrics. Its main contribution is to treat evaluation as part of the scientific process, rather than as a final check after model development.

VALID tells us what good evaluation should look like. The next challenge is determining how these principles should be implemented in practice. They should support scientific judgement rather than be reduced to a fixed checklist applied uniformly across studies. Computational biology encompasses diverse data types, experimental designs, biological questions and intended uses, so evaluation should be proportionate to the task. Clinical prediction models require rigorous evidence of out‐of‐cohort generalisability and reliability, whereas generative models used for hypothesis generation may initially be evaluated by whether they expand the plausible search space for experimental validation. Building on VALID, the community therefore needs task‐specific standards that define appropriate units of independence, realistic measures of generalisation and meaningful forms of biological validation.

The biological question should determine how methods are evaluated. For example, single‐cell methods should usually be evaluated across independent donors rather than individual cells, because cell‐level splitting can place cells from the same donor in both training and test sets. Spatial omics methods should consider tissue regions, fields of view, measurement platforms and segmentation strategies, because different segmentation approaches can introduce technical artefacts that a model may learn [2]. Protein prediction requires careful splitting based on sequence homology to prevent leakage from evolutionarily related sequences, whereas biomarker studies require validation across independent cohorts before claims of clinical utility can be made. These examples differ in detail but share the same statistical principle. Evaluation should reflect the underlying biological and experimental design rather than relying solely on abstract benchmark metrics that lack biological context [3].

The VALID framework is accompanied by a set of reflective prompts to encourage authors, reviewers and editors to think through their study design. This guidance provides an important starting point. In addition to improving reporting and assessment, the field would benefit from community‐led evaluation initiatives such as benchmarking challenges [4], living benchmark resources such as the Open Problems platform [5] and open benchmarking infrastructure such as BenchHub [6] that complements author reporting. By providing shared datasets, standardised evaluation protocols and independent comparisons, these initiatives can put VALID principles into practice more widely. They support more consistent comparisons and reduce reliance on author‐selected benchmarking metrics. They also allow benchmarks to evolve as new methods and datasets emerge, making evaluation an ongoing community resource rather than a one‐off exercise.

Khang et al. have provided an important framework for thinking about AI evaluation. The next step is to translate these principles into task‐specific standards and shared benchmarking resources. By translating VALID principles into task‐specific standards, livingbenchmarks and reusable community infrastructure, computational biology can ensure that advances in AI lead not only to better‐performing models, but also to more reliable and biologically meaningful discoveries.

## AUTHOR CONTRIBUTIONS

**Yue Cao**: Conceptualization; writing—original draft; writing—review and editing. **Jean Y. H. Yang**: Conceptualization; writing—original draft; writing—review and editing.

## CONFLICT OF INTEREST STATEMENT

Jean Y. H. Yang is one of the Editorial Board Members of *Quantitative Biology*. All authors declare no other competing interests.

## ETHICS STATEMENT

This manuscript does not involve a research protocol requiring approval by the relevant institutional review board or ethics committee.

## Data Availability

No data were generated for this article.
